# Is a colorectal neoplasm diagnosis a trigger to change dietary and other lifestyle habits for persons with Lynch syndrome? A prospective cohort study

**DOI:** 10.1007/s10689-020-00201-5

**Published:** 2020-08-08

**Authors:** Jesca G. M. Brouwer, Merel Snellen, Tanya M. Bisseling, Jan Jacob Koornstra, Hans F. A. Vasen, Ellen Kampman, Fränzel J. B. van Duijnhoven

**Affiliations:** 1 Division of Human Nutrition and Health, Wageningen University & Research, P.O. Box 17, 6700 AA Wageningen, The Netherlands; 2 Department of Gastroenterology and Hepatology, Radboud University Medical Center, Nijmegen, The Netherlands; 3 Department of Gastroenterology and Hepatology, University of Groningen, University Medical Center Groningen, Groningen, The Netherlands; 4 Department of Gastroenterology and Hepatology, Leiden University Medical Center, Leiden, The Netherlands

**Keywords:** Lynch syndrome, Diet, Smoking, Body mass index, Lifestyle, Change, Colorectal neoplasm

## Abstract

A cancer diagnosis is suggested to be associated with changes in dietary and lifestyle habits. Whether this applies to persons with familial cancer, such as Lynch syndrome (LS) is unknown. We investigated whether a colorectal neoplasm (CRN) diagnosis in persons with LS is associated with changes in dietary and lifestyle habits over time. We used data of confirmed LS mutation carriers from the GEOLynch study, a prospective cohort study. Information on dietary intake and lifestyle habits was collected with a validated semi-quantitative food frequency questionnaire and a general questionnaire administered at baseline (2006–2008) and follow-up (2012–2017). Participants’ medical records were used to identify CRN diagnoses. Changes in dietary and lifestyle habits in the CRN and the no-CRN group were compared using multivariable linear regression models for continuous variables and cross-tables with percentage change at follow-up compared with baseline for categorical variables. Of the 324 included participants, 146 developed a CRN (CRN group) between baseline and follow-up, while 178 did not (no-CRN group). Smoking cessation was more often reported in the CRN than in the no-CRN group (41.4% vs. 35.0%). There were no differences in changes of energy intake, alcohol, red meat, processed meat, dairy, fruit, vegetables and dietary fiber consumption, BMI, physical activity and NSAID use. Apart from a potentially higher likelihood of smoking cessation, we found little evidence that a CRN diagnosis is associated with changes in lifestyle habits in persons with LS.

**Electronic supplementary material:**

The online version of this article (10.1007/s10689-020-00201-5) contains supplementary material, which is available to authorized users.

## Background

It is estimated that 1 in every 279 individuals living in a Western population has a germline mutation in one of the DNA mismatch-repair (MMR) genes *MLH1*, *MSH2*, *MSH6* or *PMS2* or a deletion in the *MSH2*-adjacent *EPCAM* gene [1]. These mutations and deletions lead to Lynch syndrome (LS) [2, 3], which is the most common cause of hereditary colorectal cancer [4]. Persons with LS have an increased risk of colorectal adenomas (CRAs), and are at a high risk of developing cancer relatively early in life [3, 5–12]. In LS, colorectal cancer (CRC) is the most commonly diagnosed cancer type with cumulative risk estimates by the age of 70 years ranging from 11 to 98% [3, 11, 13–15], whereas lifetime risk in the Western population is 4–5% [16].

Apart from the mutated gene, most results of studies in persons with LS suggest that the risk of CRAs, precursor lesions of CRC [17], and CRC is increased in persons who smoke or who have a high body mass index (BMI) [18–25]. Additionally, a high alcohol consumption [23, 25, 26] and a high consumption of snack foods [27] are associated with increased risk of CRA and/or CRC. In contrast, regular physical activity [28, 29], aspirin intake [30, 31], higher fruit or fiber intakes [20], and long-term use of multivitamin and calcium supplements [32] seem to decrease CRC risk.

In the general population, it has been suggested that a cancer diagnosis may be a window of opportunity for healthy changes in diet and other lifestyle habits [33–36]. Several studies reported an increased fruit and vegetable intake, a decreased red meat intake and a high percentage of smoking cessation after a cancer diagnosis in persons diagnosed with several types of sporadic cancer [33, 35, 36]. Increases, decreases and no changes in alcohol intake, physical activity and BMI were observed [33–36]. However, not all changes in cancer-affected persons were different in comparison with changes observed in cancer-free persons [33, 35, 36].

Even though persons with LS are often diagnosed with CRAs and CRCs, i.e. colorectal neoplasms (CRNs), studies investigating whether such a diagnosis is associated with subsequent changes in diet and lifestyle habits are currently lacking in the LS population. A better understanding of changes in dietary and lifestyle factors following a CRN diagnosis in persons with LS is relevant since these changes may impact subsequent cancer risk. Therefore, our aim was to investigate whether a CRN diagnosis in persons with LS is associated with changes in dietary and lifestyle habits over time.

## Methods

### Study population

We used data of the GEOLynch study, a prospective cohort study established in the Netherlands in 2006 (ClinicalTrials.gov identifier NCT03303833) [18]. Carriers of a mutation in one of the DNA mismatch repair or *EPCAM* genes—as confirmed by a clinical genetics center—were identified through the Netherlands Foundation for the Detection of Hereditary Tumours, the Radboud University Medical Center Nijmegen and the University Medical Center Groningen, the Netherlands. Participants were between 18 and 80 years of age, Dutch-speaking, mentally competent to participate and underwent regular colonoscopy surveillance. Terminally ill patients, those living outside the Netherlands and those with familial adenomatous polyposis, inflammatory bowel diseases, and a history of proctocolectomy or colostomy were excluded.

**Fig. 1:**
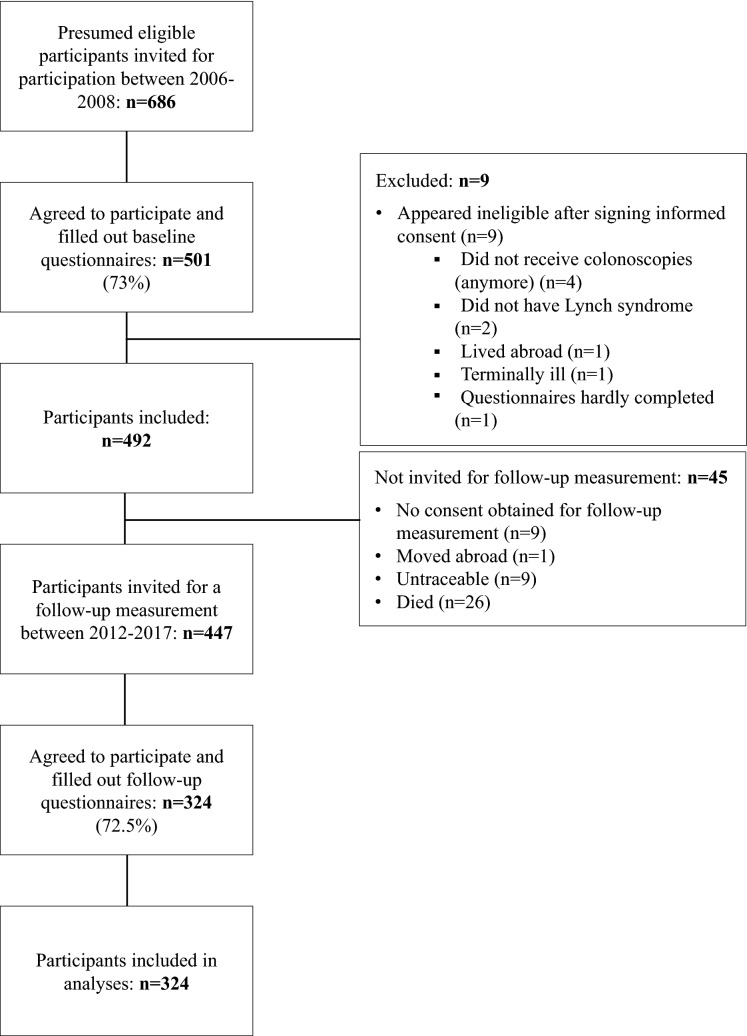
Flowchart of included study participants

A total of 686 presumed eligible subjects were invited to participate between July 2006 and July 2008 (Fig. 1). All subjects had ever received a diagnosis of Lynch syndrome. Of the 686 invited, 501 (73.0%) agreed to participate. Nine participants appeared ineligible after signing informed consent, leaving 492 included participants. All participants completed questionnaires on demographics, dietary and lifestyle characteristics at study enrolment. Considering the observational design of the study, the completed questionnaires were not used to provide participants with any personal feedback to change lifestyle habits. Between January 2012 and December 2017, 447 (90.8%) of the 492 participants were invited to complete the questionnaires again for a follow-up measurement. The remaining 45 participants were not approached since they had not given researchers consent to contact them for follow-up measurements (n = 9), were living abroad (n = 1), could not be traced (n = 9) or had died (n = 26). Of the 447 participants invited, 324 (72.5%) completed the follow-up questionnaires and were included in the current analyses. All study participants provided written informed consent and the study was approved by the Institutional Review Board CMO Region Arnhem-Nijmegen.

### Assessment of dietary intake

Habitual dietary intake of the previous month was assessed with a semi-quantitative 183-item food frequency questionnaire (FFQ). This FFQ was an updated version of two FFQ’s previously developed and validated by the department of Human Nutrition and Health, Wageningen University & Research [37, 38]. The FFQ used at baseline and follow-up were similar in terms of type of food groups and number of items per food group recalled. However, the FFQ used at follow-up contained some additional questions for the dairy food items in order to distinguish between use of fermented and non-fermented dairy products. At both time points, participants were asked to report the frequency and amount of food items used. For all items, frequencies per day and standard portion sizes were multiplied to obtain intake in grams per day. Subsequently, intake of energy and nutrients was quantified by using the Dutch food composition table (NEVO) 2011 [39]. We used the NEVO 2011 since most participants completed the follow-up FFQ around the same time period (2012). Moreover, the same (2011) version was used for both baseline and follow-up FFQ data to prevent any changes in dietary intake to be a result of using different food composition tables.

### Assessment of demographic and lifestyle characteristics

Information on age, sex, education level [low (i.e., finished primary school or lower vocational or lower general secondary education); middle (i.e., finished general secondary school, pre-university education, or vocational education); and high (i.e., finished higher professional education or university)], current height and weight, smoking status [(current, former, never) smoking of tobacco products (cigarettes, cigar, pipe)] and NSAID use [never (i.e. less than once a month) versus ever (i.e. equal to or more than once a month)] was collected through a standardized general questionnaire. Physical activity was assessed with a modified Baecke questionnaire consisting of 19 items which measures the level of physical activity in three domains: household, sports and non-sports leisure time activities [40, 41]. In accordance with the questionnaire protocol [41], each domain was scored between 1 and 5 points and domain scores were then summed to calculate the total activity score (ranging from 3 to 15), with a higher score reflecting a higher level of physical activity.

### Identification of colorectal neoplasms

Participants’ medical records were regularly reviewed (on average every 3 years) to obtain clinical information about performed colonoscopies, surgical interventions and colorectal adenomas, colorectal carcinomas and all other cancer diagnoses (excluding non-melanoma skin cancers) before recruitment and during observation time (i.e. period between baseline and follow-up questionnaire completion).

### Statistical analyses

Descriptive statistics were used to describe the characteristics at baseline for participants with and without a CRN diagnosis during observation time. Participants who were diagnosed with a CRN during observation time were included in the CRN group, while those who were not diagnosed with a CRN were included in the no-CRN group (both regardless of CRN diagnosis before baseline). Multivariable linear regression models with 95% confidence intervals (CI) were used to investigate whether changes in BMI, physical activity and each dietary variable were different for those with and without a CRN diagnosis during observation time. Analyses were adjusted for sex, age, education level, BMI (< 25.0, 25.0–30.0 and ≥ 30 kg/m^2^) and smoking status at baseline. To control for any imbalance at baseline and measurement error at baseline and follow-up, an additional adjustment for the average value of baseline and follow-up was applied for each lifestyle factor and dietary variable. For analyses of the dietary variables, a comparison was made between estimates obtained from multivariable linear regression models with and without additional adjustment for energy intake based on the residual method [42]. Since both models yielded similar findings, only the results without adjustment for energy intake were presented. The assumptions underlying the multivariable linear regression models were investigated by inspecting the models’ residuals. No violations of the assumptions were observed.

For categorical variables (smoking status, categorized BMI and NSAID use), cross-tables were created which showed the percentage of individuals in a category at follow-up for each category at baseline for the CRN and no-CRN group.

Since a CRN diagnosis before baseline may already have influenced current dietary and lifestyle habits, a sensitivity analysis was performed by repeating the analyses in participants without a CRN diagnosis before baseline only (n = 164).

A two-sided p-value of 0.05 was considered statistically significant. Data analyses were performed with the use of SAS software version 9.4 of the SAS System for Windows (SAS Institute, Cary, NC, USA).

## Results

### Participant characteristics

Of the 324 participants who completed both baseline and follow-up questionnaires, 146 (45.1%) were and 178 (54.9%) were not diagnosed with a CRN during observation time (Table 1). Participants who developed a CRN during observation time had a median age of 51.9 [interquartile range (IQR), i.e. quartile 1, quartile 3: 44.2, 57.5] years while participants without a CRN had a median age of 47.6 [IQR 38.4, 56.2] years at baseline. The majority of the participants in the CRN and no-CRN group were women (52.1% vs. 58.4% respectively). Highly educated participants accounted for 29.5% and 41.6% in the CRN group and no-CRN group respectively. At baseline 29 (19.9%) participants in the CRN group and 22 (12.4%) in the no-CRN group smoked. Overweight or obesity was seen in 65 (44.5%) participants in the CRN group and 64 (36.0%) in the no-CRN group. A median energy intake of 2134.9 [IQR 1731.0, 2622.0] kcal/day was reported in the CRN group and 2149.3 [IQR 1780.2, 2587.8] kcal/day in the no-CRN group.

**Table 1:** Characteristics of the colorectal neoplasm and no colorectal neoplasm group at baseline

	Colorectal neoplasm^a^	No colorectal neoplasm^a^
N	146	178
Age (years), median [IQR]	51.9 [44.2–57.5]	47.6 [38.5–56.2]
Mutated gene, n (%)		
MLH1	55 (37.7)	72 (40.5)
MSH2	64 (43.8)	66 (37.1)
MSH6	26 (17.8)	38 (21.4)
PMS2	1 (0.7)	2 (1.1)
Sex (woman), n (%)	76 (52.1)	104 (58.4)
Education level^b^, n (%)		
Low	47 (32.2)	43 (24.2)
Medium	56 (38.4)	61 (34.3)
High	43 (29.5)	74 (41.6)
Smoking status^a^, n (%)		
Current	29 (19.9)	22 (12.4)
Pack-years current smokers, median [IQR]	15.4 [8.0–22.5]	10.0 [1.5–16.5]
Former	67 (45.9)	77 (43.3)
Pack-years former smokers, median [IQR]	6.9 [2.9–14.5]	6.0 [2.0–11.5]
Never	48 (32.9)	75 (42.1)
BMI (kg/m^2^)^c^, median [IQR], n (%)	24.7 [23.2–26.4]	24.1 [22.3–26.4]
< 18.5	1 (0.7)	1 (0.6)
18.5–25.0	79 (54.1)	109 (61.2)
25.0–30.0	53 (36.3)	50 (28.1)
≥ 30.0	12 (8.2)	14 (7.9)
Physical activity level^d^, mean ± SD	8.4 ± 1.1	8.3 ± 1.0
Energy intake (kcal/day), median [IQR]	2134.9 [1731.0–2622.0]	2149.3 [1780.2–2587.8]
Alcohol intake (g/day), median [IQR]	10.5 [2.3–21.0]	6.5 [1.1–16.2]
Red meat intake (g/day), median [IQR]	41.3 [23.7–55.7]	40.2 [24.8–53.8]
Processed meat intake (g/day), median [IQR]	18.2 [10.7–35.2]	18.7 [7.9–32.5]
Dairy intake (g/day), median [IQR]	322.0 [220.1–458.9]	332.5 [211.7–457.9]
Fruit intake (g/day), median [IQR]	216.5 [49.7–239.3]	151.9 [78.5–230.6]
Vegetable intake (g/day), median [IQR]	137.8 [78.7–193.9]	147.7 [97.6–202.4]
Fibre intake (g/day), mean ± SD	23.7 ± 7.4	24.3 ± 7.0
NSAID use^e^, n (%)	23 (15.8)	29 (16.3)
CRN diagnosis before baseline, n (%)	78 (53.4)	82 (46.1)
Cancer other than CRC diagnosed before baseline, n (%)	23 (15.8)	27 (15.2)

The numbers reflect the information collected at baseline, unless stated otherwise. Characteristics are expressed as mean ± SD for normally distributed variables, median [IQR, i.e. quartile 1—quartile 3] for variables deviating from normality or n (%) for categorical variables

*BMI* body mass index, *CRC* colorectal cancer, *CRN* colorectal neoplasm, *IQR* interquartile range, *NSAID* non-steroidal anti-inflammatory drugs, *SD* standard deviation

^a^The CRN group includes participants with a CRN diagnosis between the baseline and follow-up measurement. If no CRN was diagnosed between baseline and follow-up, the participant was added to the no-CRN group

^b^Low reflects finishing primary school or lower vocational or lower general secondary education; middle reflects finishing general secondary school, pre-university education or vocational education; high reflects finishing higher professional education or university

^c^Percentages do not add up to 100 due to 6 missings for smoking status and 5 for BMI

^d^Physical activity level is calculated with the Baecke questionnaire [40, 41]

^e^NSAID use equal to or more than once a month

Follow-up measurements were performed after a median of 80.7 [IQR 71.4, 86.1] months after baseline measurement in the CRN group versus 82.5 [IQR 71.4, 86.5] months in the no-CRN group (data not shown). In the CRN group, a median of 2 [IQR 2, 2] CRNs per person were diagnosed during observation time. Median time between the most recently diagnosed CRN and completion of the follow-up questionnaire was 27.5 [IQR 16.7, 49.7] months. Cancer other than CRC during observation time was diagnosed in 13 (8.9%) participants of the CRN group and in 12 (6.7%) of the no-CRN group.

### Differential changes in dietary and lifestyle factors

Energy intake decreased with a mean of 295.6 ± SD 534.0 kcal/day in the CRN group and 297.2 ± 481.5 kcal/day in the no-CRN group (Table 2). The change in energy intake was not different in the CRN group compared with the no-CRN group (adjusted difference in change of − 7.5 (95% CI − 119.1, 104.0) kcal/day). Mean fruit intake decreased in the CRN group (− 15.6 ± 119.4 g/day) while it increased (4.1 ± 113.3 g/day) in the no-CRN group, but the difference in fruit intake change was not statistically significant (adjusted difference in fruit intake change of − 13.4 (95% CI − 39.7, 12.8) g/day). Changes in BMI, physical activity and other dietary intakes did not differ between the no-CRN and CRN group either.

**Table 2:** Changes in lifestyle characteristics and multivariable linear regression models for differences in change in lifestyle and dietary factors among persons with and without a CRN diagnosis

	Change per group	Crude difference (95% CI) between groups	Adjusted^a^ differences (95% CI) between groups
BMI (kg/m^2^), mean ± SD			
No CRN^b^	0.5 ± 1.7	Reference	Reference
CRN^b^	0.7 ± 2.8	0.2 (− 0.3, 0.7)	− 0.2 (− 0.5, 0.2)
Physical activity level^c^, mean ± SD			
No CRN^b^	0.3 ± 1.2	Reference	Reference
CRN^b^	0.3 ± 1.2	− 0.1 (− 0.3, 0.2)	− 0.1 (− 0.3, 0.2)
Energy intake (kcal/day), mean ± SD			
No CRN^b^	− 297.2 ± 481.5	Reference	Reference
CRN^b^	− 295.6 ± 534.0	1.5 (− 110.6, 113.7)	− 7.5 (− 119.1, 104.0)
Alcohol intake (g/day), mean ± SD			
No CRN^b^	− 1.3 ± 7.8	Reference	Reference
CRN^b^	− 1.5 ± 11.5	− 0.2 (− 2.3, 2.0)	0.3 (− 1.9, 2.5)
Red meat intake (g/day), median [IQR]			
No CRN^b^	− 9.7 [− 22.5, 3.4]	Reference	Reference
CRN^b^	− 8.1 [− 27.6, 3.0]	− 1.2 (− 6.1, 3.7)	− 0.9 (− 5.9, 4.0)
Processed meat intake (g/day), mean ± SD			
No CRN^b^	3.9 ± 25.4	Reference	Reference
CRN^b^	3.4 ± 23.7	− 0.4 (− 5.9, 5.0)	− 0.1 (− 5.5, 5.3)
Dairy intake (g/day), mean ± SD			
No CRN^b^	− 32.1 ± 212.8	Reference	Reference
CRN^b^	− 26.2 ± 159.7	5.9 (− 36.4, 48.1)	− 0.2 (− 43.3, 42.8)
Fruit intake (g/day), mean ± SD			
No CRN^b^	4.1 ± 113.3	Reference	Reference
CRN^b^	− 15.6 ± 119.4	− 19.7 (− 45.5, 6.0)	− 13.4 (− 39.7, 12.8)
Vegetable intake (g/day), median [IQR]			
No CRN^b^	− 26.2 [− 79.3, 30.5]	Reference	Reference
CRN^b^	− 15.1 [− 61.8, 14.4]	8.1 (− 8.7, 25.0)	9.4 (− 7.8, 26.7)
Fibre intake (g/day), median [IQR]			
No CRN^b^	− 2.5 [− 5.5, 1.0]	Reference	Reference
CRN^b^	− 1.0 [− 4.7, 1.3]	0.5 (− 0.9, 1.8)	0.5 (− 0.9, 1.8)

Changes are calculated among those without a missing value at both baseline and follow-up i.e. among 319 for BMI, 298 for physical activity and 318 for all dietary intakes. Changes are expressed as mean ± SD for normally distributed variables and median [IQR, i.e. quartile 1—quartile 3] for variables deviating from normality

*BMI* body mass index, *CI* confidence interval, *CRC* colorectal cancer; *CRN* colorectal neoplasm, *IQR* interquartile range, *NSAID* non-steroidal anti-inflammatory drugs, *SD* standard deviation

^a^Adjusted for age, sex, education level, BMI and smoking status at baseline and the average of baseline and follow-up intake of the corresponding dietary or lifestyle factor

^b^The CRN group includes participants with a CRN diagnosis between the baseline and follow-up measurement. If no CRN was diagnosed between baseline and follow-up, the participant was added to the no-CRN group

^c^Physical activity level is calculated with the Baecke questionnaire [40, 41]

Smoking cessation was reported by 41.4% of the smokers in the CRN group vs. 35.0% of the smokers in the no-CRN group (Table 3). A shift from overweight to normal weight was seen in 6 (11.3%) participants in the CRN group and 7 (14.0%) participants in the no-CRN group (Table 4). In the CRN group, 10.3% increased the use of NSAIDs from less than once a month to equal to or more than once a month against 12.1% in the no-CRN group (data not shown).

**Table 3:** Smoking behaviour at baseline and at follow-up time by subgroup

	Smoking status at follow-up		
	Current	Former	Never
No colorectal neoplasm^a^			
Smoking status at baseline			
Current (N = 20)	13 (65.0)	7 (35.0)	0 (0.0)
Former (N = 75)	5 (6.7)	70 (93.3)	0 (0.0)
Never (N = 75)	0 (0.0)	3 (4.0)	72 (96.0)
Colorectal neoplasm^a^			
Smoking status at baseline			
Current (N = 29)	17 (58.6)	12 (41.4)	0 (0.0)
Former (N = 64)	1 (1.6)	63 (98.4)	0 (0.0)
Never (N = 48)	0 (0.0)	3 (6.3)	45 (93.8)

Percentages of those without missing values in smoking status. Reported values reflect n (%). *CRN* colorectal neoplasm

Participants who reported to be current smoker at baseline and never smokers at follow-up (n = 2) or to be former smoker at baseline and never at follow-up (n = 5) were not taken into account

^a^The CRN group includes participants with a CRN diagnosis between the baseline and follow-up measurement. If no CRN was diagnosed between baseline and follow-up, the participant was added to the no-CRN group

**Table 4:** Body mass index (BMI) at baseline and at follow-up time by subgroup

	BMI (kg/m^2^) at follow-up^b^			
	Underweight	Normal weight	Overweight	Obese
No colorectal neoplasm^a^				
BMI (kg/m^2^) status at baseline^b^				
Underweight (N = 1)	0 (0.0)	1 (100.0)	0 (0.0)	0 (0.0)
Normal weight (N = 109)	2 (1.8)	84 (77.1)	23 (21.1)	0 (0.0)
Overweight (N = 50)	0 (0.0)	7 (14.0)	37 (74.0)	6 (12.0)
Obese (N = 14)	0 (0.0)	0 (0.0)	2 (14.3)	12 (85.7)
Colorectal neoplasm^a^				
BMI (kg/m^2^) status at baseline^b^				
Underweight (N = 1)	1 (100.0)	0 (0.0)	0 (0.0)	0 (0.0)
Normal weight (N = 79)	1 (1.3)	67 (84.8)	10 (12.7)	1 (1.3)
Overweight (N = 53)	0 (0.0)	6 (11.3)	40 (75.5)	7 (13.2)
Obese (N = 12)	0 (0.0)	0 (0.0)	2 (16.7)	10 (83.3)

Percentages of those without missing values in BMI. Reported values reflect n (%)

*BMI* body mass index, *CRN* colorectal neoplasm

^a^The CRN group includes participants with a CRN diagnosis between the baseline and follow-up measurement. If no CRN was diagnosed between baseline and follow-up, the participant was added to the no-CRN group

^b^Underweight reflects a BMI < 18.5 kg/m^2^, normal weight a BMI of 18.5 to 25.0 kg/m^2^, overweight a BMI of 25.0 to 30.0 kg/m^2^ and obese a BMI ≥ 30 kg/m^2^

### Sensitivity analyses

Participants diagnosed with a CRN before baseline (n = 160) were excluded in the sensitivity analysis. Of the 164 participants without a CRN diagnosis before baseline, 68 (41.5%) developed a CRN during observation time while 96 (58.5%) did not. The difference in percentage of smoking cessation between the CRN and no-CRN group was larger compared with that in all participants with smoking cessation reported by 6 (75.0%) of the 8 smokers at baseline in the CRN group and 3 (25.0%) of the 12 smokers at baseline in the no-CRN group (Supplemental table S1). Differences in changes in physical activity, BMI, dietary intakes and NSAID use between the CRN and no-CRN group tended to be larger than in the main analyses involving all participants for most habits but remained statistically non-significant for all (Supplemental table S2 and S3).

## Discussion

We investigated whether a CRN diagnosis is associated with changes in dietary and lifestyle habits in persons with LS. Apart from a potentially higher likelihood of smoking cessation, we found little evidence for an association between a CRN diagnosis and changes in dietary and lifestyle habits in persons with LS.

To date, studies investigating the role of dietary and lifestyle factors in LS-associated cancer risk have mainly focused on the association between diet and lifestyle and subsequent incidence of CRAs and (colorectal) cancer. Though such studies are of obvious importance, we sought a different and more novel approach by investigating the impact of a CRN diagnosis on subsequent changes in dietary and lifestyle factors in persons with LS over time. In the general population, it has been suggested that a cancer diagnosis may be a window of opportunity for healthy changes in diet and other lifestyle habits [33–36]. Several studies reported an increased fruit and vegetable intake, a decreased red meat intake and a decrease in BMI after a cancer diagnosis [34–36]. We did not observe this in our population of persons with LS. This may be explained by the high percentage of colorectal adenomas (89.0%) instead of carcinomas in the CRN group. Colorectal adenomas, precursor lesions of CRC, that are identified during surveillance colonoscopy are removed before they can progress into CRC. Therefore, it could be speculated that an adenoma, which is directly removed after identification without any additional treatment, will have less impact on diet and lifestyle as compared to a CRC or cancer diagnosis. However, due to the small numbers of CRC (n = 16) and cancer cases (n = 35) in our cohort, it was not possible to further study changes in dietary and lifestyle habits in these cancer-affected subgroups. Hence, a possible differential impact of a (colorectal) cancer diagnosis as compared with an adenoma diagnosis on changes in dietary and lifestyle habits in persons with LS could not be eliminated in this study.

Despite the absence of an association between CRN diagnosis and changes in most dietary and lifestyle habits in our population, we did observe a higher percentage of smoking cessation in those with a CRN than in those without a CRN. This result was even stronger when the analyses were repeated in participants without a CRN diagnosis before baseline only. Similar findings have been observed for cancer-affected vs. cancer-free persons in studies among the general population [33, 36]. It should however be mentioned that in our study the number of participants in the subgroups of smoking status (e.g. number of current smokers who quit smoking was n = 12 in the CRN and n = 7 in the no-CRN group) was too small to allow statistical adjustment for other factors that may potentially influence a change in smoking behavior (e.g. age). The differences in percentage of smoking cessation observed in our population may therefore be explained by other factors than a CRN diagnosis, so the results need to be interpreted with caution. Still, our findings carefully suggest that a CRN diagnosis might trigger smoking cessation in persons with LS.

Our study has some limitations which should be considered. First, although this study is one of the largest prospective cohort studies in persons with LS worldwide, it is a small study compared to studies in the general population. As a result, we had limited power to detect differences in change, particularly for categorical variables (i.e. smoking status and BMI categories) between those with and without a CRN diagnosis or to do sub-analyses (e.g. to investigate differences in change between those with multiple CRNs and those without CRNs). Second, we relied on self-reported measures of dietary and lifestyle factors, which may be subject to recall bias to promote social desirability. However, if social desirable answers were given, it is not likely to have affected those with and without a CRN diagnosis differently. Third, information on dietary and lifestyle habits was collected at a median of 27.5 months after the most recent CRN diagnosis during observation time. Hence, it is possible that in our study short-term changes in diet and lifestyle were missed but long-term changes could still be captured. Nevertheless, previous studies reporting on changes in diet and lifestyle after a cancer diagnosis in the general population had similar [33, 36], or even longer [34] lengths of follow-up since diagnosis. We therefore do not expect that time since CRN diagnosis has had much impact on our results. Fourth, participants in the CRN group were on average more likely to have had a pre-baseline CRN diagnosis (53.4%) as compared to those in the no-CRN group (46.1%). Since a CRN diagnosis before baseline may already have influenced dietary and lifestyle habits, we conducted a sensitivity analysis by repeating the analyses in participants without a CRN diagnosis. Still, our findings remained non-significant and in the same direction as compared to results of the main analyses involving all participants. Therefore, we do not expect that the difference in proportion of participants with a pre-baseline CRN diagnosis between the CRN and the no-CRN group has substantially influenced our results. In addition, although all participants had been aware of their LS diagnosis before study inclusion, we do not know when participants became aware of their LS status. It could be hypothesized that a diagnosis of a genetically inherited syndrome may trigger a change in dietary and lifestyle habits and that this change already occurred before our study inclusion. A study by Ramsey et al. [43] found that hypothetical testing for a gene variant predisposing to CRC increased participants’ motivation to adopt healthier diet and exercise behaviors. A similar finding was observed by Brodersen et al. [44]. In that study, first degree relatives of CRC patients at high risk of CRC, based on hypothetical genetic test results, more often anticipated leading a healthier lifestyle compared to those at low risk. Nevertheless, an increased motivation for behavioral change, as found in these studies, does not necessarily imply changes will occur. For instance, Kim et al. [45] found that LS mutation carriers who discovered their genetic predisposition to CRC were not more likely to quit smoking compared to LS carriers who did not obtain their genetic test results. Moreover, in a qualitative study among a population similar to ours, Visser et al. [46] found that receiving a LS diagnosis was not reported as an important determinant of adherence to lifestyle recommendations and was actually found to be a barrier in adapting to a more healthy lifestyle. We therefore expect that the LS diagnosis has had little to no effect on our results. A final consideration relates to the generalizability of our study sample. Participants were recruited via a hereditary cancer registry and hospitals and were therefore more likely to originate from LS families with the highest risk of cancer. It may hence not be a random sample of the total LS population. Generalizing the findings to all LS mutations carriers might therefore not hold.

Strengths of this study include the prospective and longitudinal design which enabled us to investigate changes in dietary and lifestyle habits over time. Moreover, we were able to collect detailed data on a wide range of modifiable risk factors which are associated with many cancer types in the general population.

In conclusion, apart from a potentially higher likelihood of smoking cessation, we found little evidence that a CRN diagnosis is associated with changes in dietary and lifestyle habits in persons with LS. The growing evidence that a healthy diet and lifestyle may modify LS-associated cancer risk highlights the need to identify effective support for health behavior change in persons with LS.

## Electronic supplementary material

Below is the link to the electronic supplementary material.

Supplementary file1 (PDF 181 kb)
