# Supplementary material for: Is a colorectal neoplasm diagnosis a trigger to change dietary and other lifestyle habits for persons with Lynch syndrome? A prospective cohort study
Source: Fam Cancer. 2020 Aug 8;20(2):125–35. doi: 10.1007/s10689-020-00201-5 (PMC8064993; doi:10.1007/s10689-020-00201-5)
Supplement: Supplementary file 1 — Supplementary file1 (PDF 181 kb) [file 10689_2020_201_MOESM1_ESM.pdf]

Supplemental tables

Supplemental table S1. Sensitivity analysis excluding those with a colorectal neoplasm diagnosis before baseline. Smoking behaviour at baseline and at follow-up time by subgroup.[*]

		Smoking status at follow-up		
**No colorectal neoplasm$^{\alpha}$**		Current	Former	Never
Smoking status at baseline	Current (N=12)	9 (75.0)	3 (25.0)	0 (0.0)
	Former (N=41)	3 (7.3)	38 (92.7)	0 (0.0)
	Never (N=40)	0 (0.0)	0 (0.0)	40 (100.0)
**Colorectal neoplasm$^{\alpha}$**		Current	Former	Never
Smoking status at baseline	Current (N=8)	2 (25.0)	6 (75.0)	0 (0.0)
	Former (N=30)	0 (0.0)	30 (100.0)	0 (0.0)
	Never (N=29)	0 (0.0)	2 (6.9)	27 (93.1)

[*]Percentages of those without missing values in smoking status. Reported values reflect n (%). One participants who reported to be a former smoker at baseline and a never smoker at follow-up was not taken into account. $^{\alpha}$The colorectal neoplasm group includes participants with a colorectal neoplasm diagnosis between the baseline and follow-up measurement. If no colorectal neoplasm was diagnosed between baseline and follow-up, the participant was added to the no colorectal neoplasm group.

Supplemental table S2. Sensitivity analysis excluding those with a colorectal neoplasm diagnosis before baseline. Changes in lifestyle characteristics and multivariable linear regression models for differences in change in lifestyle and dietary factors among before baseline in those with and without a CRN diagnosis during follow-up.[α]

	**Change per group**	**Crude difference (95%CI) between groups**	**Adjusted[ß] differences (95%CI) between groups**
BMI (kg/m^2), mean ± SD			
No CRN[¥]	0.4 ± 1.9	Reference	Reference
CRN[¥]	0.3 ± 1.8	-0.2 (-0.8, 0.4)	-0.1 (-0.6, 0.4)
Physical activity level[¶], mean ± SD			
No CRN[¥]	0.3 ± 1.2	Reference	Reference
CRN[¥]	0.4 ± 1.3	0.1 (-0.3, 0.5)	-0.1 (-0.4, 0.3)
Energy intake (kcal/day), mean ± SD			
No CRN[¥]	-334.6 [-613.7, -11.9]	Reference	Reference
CRN[¥]	-286.7 [-590.4, -159.7]	-89.2 (-255.2, 75.9)	-33.9 (-195.7, 128.0)
Alcohol intake (g/day), mean ± SD			
No CRN[¥]	-2.0 ± 7.1	Reference	Reference
CRN[¥]	-1.0 ± 8.7	1.0 (-1.4, 3.5)	1.4 (-1.2, 4.0)
Red meat intake (g/day), median [IQR]			
No CRN[¥]	-8.2 [-22.0, 4.2]	Reference	Reference
CRN[¥]	-9.4 [-29.3, 1.8]	-3.6 (-10.6, 3.3)	-4.6 (-11.5, 2.4)

Processed meat intake (g/day), mean ± SD			
No CRN¥	4.9 ± 22.5	Reference	Reference
CRN¥	4.9 ± 24.0	0.0 (-7.3, 7.3)	-0.2 (-7.2, 6.7)
Dairy intake (g/day), mean ± SD			
No CRN¥	-52.7 ± 208.9	Reference	Reference
CRN¥	-48.2 ± 149.7	4.2 (-54.8, 63.1)	6.1 (-55.7, 67.9)
Fruit intake (g/day), mean ± SD			
No CRN¥	22.0 ± 105.6	Reference	Reference
CRN¥	1.8 ± 131.0	-20.2 (-57.1, 16.7)	-22.7 (61.9, 16.4)
Vegetable intake (g/day), median [IQR]			
No CRN¥	-25.4 [-82.1, 28.0]	Reference	Reference
CRN¥	-14.3 [-55.6, 27.6]	13.7 (-10.6, 38.1)	20.6 (-4.0, 45.2)
Fibre intake (g/day), median [IQR]			
No CRN¥	-2.3 [-5.8, 1.6]	Reference	Reference
CRN¥	-1.1 [-4.8, 1.1]	-0.1 (-2.2, 1.9)	0.5 (-1.5, 2.5)

αChanges are calculated among those without a missing value at both baseline and follow-up i.e. among 161 for BMI, 153 for physical activity and 161 for all dietary intakes. Changes are expressed as mean ± SD for normally distributed variables and median [IQR, i.e. quartile 1 – quartile 3] for variables deviating from normality. ßAdjusted for age, sex, education level, BMI and smoking status at baseline and the average of baseline and follow-up intake of the corresponding dietary or lifestyle factor. ¥The CRN group includes participants with a CRN diagnosis between the baseline and follow-up measurement. If no CRN was diagnosed between baseline and follow-up, the participant was added to the no-CRN group. ¶Physical activity level is calculated with the Baecke questionnaire [1,2]. BMI, body mass index; CI, confidence interval; CRC, colorectal cancer; CRN, colorectal neoplasm: IQR, interquartile range: NSAID, non-steroidal anti-inflammatory drugs: SD, standard deviation.

Supplemental table S3. Sensitivity analysis excluding those with a colorectal neoplasm diagnosis before baseline. Body mass index (BMI) at baseline and at follow-up time by subgroup.[*]

		BMI (kg/m^2) at follow-up[¶]			
**No colorectal neoplasm[α]**		Underweight	Normal weight	Overweight	Obese
BMI (kg/m^2) status at baseline[¶]	Underweight (N=0)	-	-	-	-
	Normal weight (N=62)	2 (3.2)	46 (74.2)	14 (22.6)	0 (0.0)
	Overweight (N=28)	0 (0.0)	5 (17.9)	19 (67.9)	4 (14.3)
	Obese (N=4)	0 (0.0)	0 (0.0)	1 (25.0)	3 (75.0)
**Colorectal neoplasm[α]**		Underweight	Normal weight	Overweight	Obese
BMI (kg/m^2) status at baseline[¶]	Underweight (N=1)	1 (100.0)	0 (0.0)	0 (0.0)	0 (0.0)
	Normal weight (N=40)	1 (2.5)	34 (85.0)	5 (12.5)	0 (0.0)
	Overweight (N=22)	0 (0.0)	2 (9.1)	17 (77.3)	3 (13.6)
	Obese (N=4)	0 (0.0)	0 (0.0)	1 (25.0)	3 (75.0)

[*]Percentages of those without missing values in BMI. Reported values reflect n (%). [α]The CRN group includes participants with a CRN diagnosis between the baseline and follow-up measurement. If no CRN was diagnosed between baseline and follow-up, the participant was added to the no-CRN group. [¶]Underweight reflects a BMI<18.5 kg/m^2, normal weight a BMI of 18.5 to 25.0 kg/m^2, overweight a BMI of 25.0 to 30.0 kg/m^2 and obese a BMI≥30 kg/m^2. BMI, body mass index; CRN, colorectal neoplasm.
